# The specificity and the development of social-emotional competence in a multi-ethnic-classroom

**DOI:** 10.1186/1753-2000-3-16

**Published:** 2009-05-28

**Authors:** Katja Petrowski, Ulf Herold, Peter Joraschky, Agnes von Wyl, Manfred Cierpka

**Affiliations:** 1 Dept of Psychotherapy & Psychosomatic Medicine, University Hospital Carl Gustav Carus, University of Technology Dresden, Fetscherstr. 74, 01307 Dresden, Germany; 2 University of Basel, Department of Child and Adolescent Psychiatry, Schaffhauserrheinweg 55, CH – 4058 Basel, Switzerland; 3 Dept of Psychosomatic Medicine Research and Family Therapy University of Heidelberg, Bergheimer Strasse 54, 69115 Heidelberg, Germany

## Abstract

**Background:**

Ethnic diversity in schools increases due to globalization. Thus, the children's social-emotional competence development must be considered in the context of a multi-ethnic classroom.

**Methods:**

In this study, the social-emotional competence of 65 Asian-American and Latin-American children was observed at the beginning and the end of their kindergarten year.

**Results:**

Initially, significant differences existed among these ethnic groups in respect to moral reasoning. Furthermore, the male children showed more dysregulated aggression but the female children implemented more moral reasoning than their male counterparts. These ethnic specificities did not disappear over the course of the year. In addition, a significant change in avoidance strategies as well as expressed emotions in the narrative took place over the course of one year.

**Conclusion:**

Ethnic specificity in social-emotional competence does exist independent of gender at the beginning as well as at the end of the kindergarten year in a multi-ethnic kindergarten classroom.

## Introduction

Multi-ethnic kindergarten classrooms can be found in many parts of the world, but especially in the USA [1]. Since social development is based on observation and imitation in respect to the social learning theory [2], and the child's peers play a role in social development [3], the influence of the multi-ethnic classroom on social-emotional competence must be taken into serious consideration.

Behaviorism and the social learning theory explained the social-emotional development through processes such as reinforcement, punishment, conditioning, observations and limitations [2,4,5]. This development took place at the preschool/kindergarten age (effect sizes of the increase in social-emotional competence = .24 and .33, [6,7]), not only inside but also outside the family [2]. Therefore, exposure to prosocial or antisocial peers was related to changes in social behavior over the course of the preschool/kindergarten year [3,8]. Furthermore, the development from age two to five was crucial for the manifestation of early childhood aggression [9,10] as well as the development of emotion and self-regulation which prevented early childhood aggression [11,12]. Children of this age primarily tend to use hedonistic reasoning or needs-oriented (primitive empathetic) social reasoning [13]. However, the development of social competence was affected by the varying degree of the normativity of the society and subcultural variations [14].

Individualism and collectivism were possible antecedent values in societies for the explanation of social-emotional development [15]. Individualism was more prevalent in Western societies than in the more traditional societies of developing countries, where collectivism was the dominant value [e.g. [16,17]]. An orientation of a society toward individualism, on the one hand, or collectivism, on the other, implied basic psychological functioning such as the expression of emotions, moral reasoning, the style of conflict resolution and social competence.

The importance of social responsibility and moral reasoning differed across cultures and subcultures. In the USA, interpersonal responsiveness and caring was viewed as less obligatory and more of a personal choice [18]. European-American and Mexican-American children did not differ in regard to the degree of obligation to the family. However, European-American children equated obligation to the family with relationship quality and closeness to family whereas to Mexican-American children obligation to the family and to the collective was a part of being a family or a group member [19].

Collectivism and individualism also influenced collaboration and conflict resolution style. European-American children high in individualism preferred confronting others and immediately taking a turn rather than waiting for a turn in group interaction tasks [20,21]. Furthermore, they communicated and resolved conflicts in an individualistic mode. In contrast, Mexican-American children high in collectivism preferred accommodation as a mode of handling conflicts with family and friends [22]. European-American children were less likely to use equality norms in interactions with in-group members than Chinese children [23].

Focusing on social competence, socialization not only inside but also outside the family and its cultural background must be considered based on the social learning theory [2]. In the social-emotional and moral development empathy played an essential part of social behavior [24,25]. According to the "main effect model" [26] empathy inhibited aggression and anger, which was connected positively to emotional expressions and essential for further development [27]. Mexican-American and European-American children did not differ in sharing candy with a classmate [28]. However, Mexican-American children were generally more inclined to share something with a peer than European-American children were [29,30]. Furthermore, in Chinese kindergarten classrooms the incidences of sharing and comforting were higher than in American kindergarten classrooms [31] since Chinese societies generally emphasized responsibility and prosocial behavior towards others [32]. Based on the literature, ethnically specific social-emotional development can be assumed even for the subcultures of a country.

Concerning multi-ethnic classrooms, the socialization within the family and the peers in the preschool/kindergarten class may augment or counteract cultural influences. However, results on the reciprocal influence of the ethnic background and the peers on child development are not yet available. Only one study evaluated the status quo and found ethnic differences in normative beliefs, expressed emotions and interpersonal conflicts in the multi-ethnic classroom [33]. Latin-American children reported higher levels of normative beliefs about aggression and expressed more aggressive fantasies but reported less fights than African-American children did [33]. However, we assume that these cultural differences concerning aggression are dependent of gender specificities as some of the most well-supported findings in the research literature showed that boys were more aggressive than girls [34-36]. Also, there are no data available on how the social-emotional competencies develop in respect to ethnic backgrounds over the course of a year.

Based on the literature, the following hypotheses can be stated:

1. It can be presumed that the different ethnic groups in the classroom differ in the degree of social-emotional competence at the beginning of the kindergarten year. The Latin-American children will more likely show more moral reasoning, more expressed emotions and less interpersonal conflicts than the Asian-American children.

2. By the same token, at the end of kindergarten year the two ethnic groups may display the same level of moral reasoning, more expressed emotions and less interpersonal conflicts due to the reciprocal adaptation.

3. At both, the beginning and the end of the kindergarten year, the boys may show more aggressive behavior than the girls.

4. Furthermore, the social-emotional competence in this age group may increase during the course of the year. Hereby, the two ethnic groups may develop differently, depending on their initial level.

Detailed assessment of social-emotional competence in a multi-ethnic classroom would present an interesting scope. The USA as a country that attracts large numbers of immigrants from all over the world, seem predestinated for conducting studies in the multi-ethnic classroom [37]. Since the preschool/kindergarten age is the sensitive age-range for the development of social-emotional competence and kindergarten is a child's first official contact with the American school system, the kindergarten year was chosen for evaluating social-emotional competence and its development over the course of one year.

## Method

The study was conducted in four kindergarten classes located in Oakland, California, USA. These kindergarten classes were chosen due to their high ethnic diversity. All of the children in these classes were the offspring of parents who had been brought up in their country of origin, later immigrating to the USA (first generation immigrants). Among them, there was a large group of Asian-American and Latin-American children. The parents of the children of these Oakland kindergarten classes were asked to give written consent to allow their children to participate in the study. In the first test, at the beginning of the kindergarten year, 65 of 90 (84%) children, and in the second test, at the end of the kindergarten year, 65 of the original 80 (95%) children were tested, respectively. The remaining children were Afro-American children or the parents did not agree on the study participation. Children who were not tested at the beginning as well as at the end of the kindergarten year were not considered in the calculations. The first test was conducted in August/September, at which time all of the children had reached the age of five (31 by August). The second test took place in May of the following year. The school year as such proceeded as in any normal public classroom in the USA does.

The sample consisted of 65 children of which 30 were female and 35 were male. In this sample 46 percent were Asian-Americans (N = 27) from China, 53 percent Latin-Americans (N = 38) from Mexico. This was not a representative sample, the small sample sizes being due to the distribution in this kindergarten. The school board did not permit the assessment of detailed information of the sample concerning socio-demographic characteristics such as income, family situation, year of immigration and religion. The distribution of the social-emotional behavior at the beginning and at the end of the kindergarten year is presented for the male and female children of the different ethnic groups in Table 1.

**Table 1:** Ethnic and gender specificity at the beginning of the kindergarten year

	**At the beginning**	**At the end**	**At the beginning of the kindergarten year**				**At the end of the kindergarten year**			
**Variable**	**M (SD)**	**M (SD)**	**M(SD)** **Asian-American**		**M(SD)** **Latin-American**		**M(SD)** **Asian-American**		**M(SD)** **Latin-American**	

	**N = 65**	**N = 65**	**male n = 17**	**female n = 10**	**male n = 18**	**female n = 20**	**male n = 17**	**female n = 10**	**male n = 18**	**female n = 20**

**Empathetic relation**	7.81 (2.39)	6.96 (2.77)	7.95 (2.34)	7.46 (2.78)	6.71 (1.42)	8.44 (2.34)	5.75 (2.63)	7.42 (4.37)	7.47 (1.81)	7.76 (3.18)
**Avoidance strategies**	12.09 (5.83)	12.82 (6.44)	11.39 (5.85)	10.65 (3.40)	12.56 (5.20)	12.65 (6.47)	14.67 (6.01)	11.97 (6.14)	12.22 (6.63)	11.90 (6.69)
**Moral themes**	14.58 (5.20)	15.07 (5.09)	10.90 (3.99)	14.75 (4.44)	14.14 (4.04)	17.58 (4.45)	12.21 (5.13)	15.00 (5.29)	14.54 (5.20)	17.79 (4.16)
**Interpersonal conflict**	1.11 (1.39)	0.87 (1.08)	0.48 (0.52)	0.80 (1.03)	1.52 (1.67)	1.31 (1.50)	0.83 (1.03)	1.05 (1.27)	0.46 (0.64)	1.12 (1.32)
**Dissociation codes**	1.45 (2.37)	1.26 (2.09)	1.37 (1.91)	0.41 (0.71)	2.60 (3.47)	0.87 (1.46)	1.24 (1.89)	0.87 (0.71)	1.58 (2.68)	0.92 (1.64)
**Dysregulated aggression**	6.77 (9.41)	5.75 (8.54)	8.92 (9.43)	2.74 (3.55)	10.43 (12.48)	3.38 (5.93)	7.80 (8.49)	2.32 (1.26)	7.09 (10.39)	2.49 (5.07)
**Narrative emotions**	6.73 (4.63)	9.13 (5.35)	7.68 (5.00)	6.16 (3.60)	7.33 (4.78)	5.41 (3.97)	10.24 (5.08)	10.37 (3.60)	9.11 (5.85)	7.02 (5.16)

Since the aim was to observe social-emotional behavior in the doll play at the beginning and the end of the kindergarten year, the Mac Arthur Story Stem Battery (MSSB) was implemented [38,39]. The Mac Arthur Story Stem Battery is a method [38,39] that reveals the inner world and representations of young children of age three and older (developed by Emde, Bretherton and colleagues; cf. [40-43]). This method bases on a standardized sequence of the beginnings of stories like short, half-structured conflict situations. The interviewer starts by telling and playing the beginning of one story with puppets including a conflict. Then, the child is being asked to show and to tell what happens next in this story. These 30 minute-interviews were video-taped and coded in respect to the different content and behavior addressed in the coding manual. The MSSB is a valid and reliable instrument (for further details see [39]).

In this study, ten stories were chosen according to the targeted social-emotional competence to be tested (one warm-up story and nine conflict stories): Susan/George's Birthday (warm up story), The Hot Soup, Barney's Disappearance, The Departure, The Return of the Parents, The Lost Key, The Exclusion, The Mother's Headache, Three is a Crowd, and The Sand Castle. The warm up story is a beginning of a conflict story as are the other stories. With this warm-up story the child gets accommodated to the setting and the procedure so that the child can follow through the following stories without any assistance. Each child completed the nine narratives from the MacArthur Story-Stem Battery (Table 2 presents a brief description of each story, original stories and examples described by [41]).

**Table 2:** Survey of the administered MSSB stories described by Warren et al. (1996).

0)	The Birthday of Susanne/George (warm-up story): The family celebrates the birthday of Susanne/George.
1)	The Hot Soup: Although the mother had forbidden it, the child grasps at the pot with the hot soup, pouring it out and burning her hand.
2)	Barney's Disappearance: The child goes to the garden for playing with the dog Barney, but Barney is not there.
3)	The Departure: The parents drive on a trip overnight and the children remain with the grandmother.
4)	The Return of the Parents: The parents return from their trip.
5)	The Lost Key: The child enters the room and hears mother and father arguing over a lost key.
6)	The Exclusion: Mother and father want to be alone and send the child to its room to play.
7)	Mother's Headache: The mother has a headache and asks Susanne/George to switch off the television. There the friend comes by and absolutely wants to watch television.
8)	Three is a Crowd: The child and the friend play with his new ball. There comes the small brother from the house and wants to join in, but the friend doesn't want that at all.
9)	The Sand Castle: A small child in a park built a sand castle. The friend says to the child: Come on, we break the little guy's sand castle.

**Instruction after playing the respective scene**: "*Show and tell, what happens next!"*

The children's play narratives were videotaped in a playroom laboratory and coded with the MacArthur Narrative Coding System [44] (for further details of the coding manuals see [43,45,46]). – The author attended a training which was provided by the original author of the coding manual JoAnn Robinson. JoAnn Robinson developed and validated the coding system and published it [43]. In this coding manual, the social-emotional competence was evaluated by several additional global scales besides those of empathetic relation and dysregulated aggression. Thus, the social problem-solving can be analyzed in more detail in order to specify strategies besides aggression. The following content themes expressed by the children's stories were coded (global scales): Empathic relation, avoidance strategies, moral themes, interpersonal conflict, dysregulated aggression, dissociation codes and narrative emotions (see [46]). The content themes were coded as 'present' or 'absent'. Under interpersonal conflict, the aspects of competition, rivalry/jealousy, exclusion, active refusal of empathy/help and verbal conflict were summarized. For empathetic relation in the stories, sharing, empathy/helping, affiliation and affection had to be present. To show avoidance strategies, the doll play had to include self-exclusion, repetition, denial, passive refusal of empathy, sudden sleep onset and sensomotor/mechanical preoccupation. Moral themes were coded if scenes included non-/compliance, shame, blame, teasing/taunting, dishonesty, punishment/discipline, reparation/guilt and politeness. Aggressive themes were coded if the subject engaged in physically aggressive acts directed toward another character, prop, or object by the subject or the puppet character. Such interactions had a negative quality to them and included hostile or destructive gestures and forms of physical violence such as an object being thrown at another character with the intent to cause pain. Personal injury was coded whenever there was an instance of a character's being physically hurt or injured. The focus needed to be on the injury or pain and not just on the act of aggression itself. Atypical negative responses were coded for any atypical or disorganized response with a negative tone. The narrative emotion code summarizes all expressed emotions in the narrative.

The doll play was conducted by a trained interviewer. The stories and the actual doll play behavior of the children were transcribed from the tape and blind-coded. Coding was carried out by two especially trained individuals. The inter-rater agreement was calculated based on 20 children out of the original 80 children (9 stories with 7 scales per child respectively). Herefore, each rating-scale (7 of them) was added up for the nine stories of a child in order to gain seven general ratings for this child. For the inter-rater agreement Kappas were calculated for the 7 global scales for each of the 20 children at the two measurement points. Hereby, the inter-rater agreement ranged between Kappa = .74 to .82. In the case of disagreement between the two reliable rater a consensus rating with a third reliable rater was implemented.

For the statistical analysis, SPSS.15.0 was used.

Hypothesis 1 +2+3: Ethnic differences in social-emotional competence at the beginning and at the end of the kindergarten year:

A two factorial (gender, ethnic group) multivariate analysis of variance was calculated to compare the ethnic groups at the beginning of the kindergarten year. The same procedure was chosen to compare the ethnic groups at the end of the kindergarten year. Since the gender had an influence on the social-emotional competence, and the gender distribution in the ethnic groups was diverse, the gender needed to be considered in the calculations as a second factor. Regarding the accumulation of error effects the statistical program SPSS automatically corrected the range of significance and the usually used range of values can be applied (p < 0.05). For the multivariate overall effect of ethnicity Wilks-Lambda was calculated. For the scale-specific effects the corrected model was used in order to minimize the effect of the sample size. In the Tables 2 and 3 the interaction effect of ethnicity and gender is not displayed since there are no significant effects.

Hypothesis 4 – ethnic differences in social-emotional competence over the development of one year:

To determine the changes over the course of one year, a three-factorial (time, gender, ethnic group) univariate analysis of variance with repeated measurement points was calculated separately for each scale of the MSSB. Greenhouse-Geisser correction was applied to account for violation of the sphericity assumption. In the Table 5 the interaction effect of ethnicity and gender is not displayed since there are no significant effects.

## Results

The first consideration concerned the ethnic and gender differences in social-emotional competence at the beginning and the end of the kindergarten year. Later on, the changes in the social-emotional competence were focused on in order to evaluate ethnic specific development.

Beginning of the kindergarten year:

In general the two ethnic groups differed significantly in their social-emotional competence at the beginning of the kindergarten year (see Table 3). In Table 3 a two factorial (gender, ethnic group) multivariate analysis of variance is displayed which shows the significant effects of the multiple comparisons with corrections for the error effects. The descriptive statistics of the MSSB codes are presented in Table 1. Since there was no significant interaction of gender and ethnicity these effects were not displayed in the Table. Comparing the two ethnic groups, the Latin-American children most frequently utilized a moral approach while the Asian-American children employed this method the least.

In general the gender differed significantly in their social-emotional competence at the beginning of the kindergarten year (see Table 3). The female children included more moral themes in their play than the male children did. The most dysregulated aggression and dissociation codes were employed by the male children. Therefore, the two ethnic groups and the different genders entered the kindergarten year with small differences in social-emotional competence.

**Table 3:** Ethnic and gender specificity at the beginning of the kindergarten year (multivariate two factorial analysis of variance).

	**F (df = 1;2)^d^**		**p (F)**				**Wilks-Lambda^c^**		**p**	
**Variable**	**ethnic**	**gender**	**ethnic**	**gender**	**η^2^_ethnic_^a^**	**η^2^_gender_^b^**	**ethnic**	**gender**	**ethnic**	**gender**

							0.78	0.71	**.04**	**<.01**
**Empathetic relation**	0.03	0.79	.38	.47	<.01	.02				
**Avoidance strategies**	1.17	0.18	.29	.67	.02	<.01				
**Moral themes**	5.29	6.20	**.03**	**.02**	.11	.15				
**Interpersonal conflict**	2.55	0.13	.12	.72	.08	<.01				
**Dissociation codes**	1.18	3.80	.28	**.05**	.04	.09				
**Dysregulated aggression**	0.01	5.48	.92	**.02**	<.01	.13				
**Narrative emotions**	1.06	0.32	.31	.57	<.01	.04				

^a, b^Effect sizes for the ethnic groups and gender specific change; ^c ^Exact test; ^d ^corrected model; Ethnic*gender interaction not significant

End of the kindergarten year:

In general the two ethnic groups differed significantly in their social-emotional competence at the end of the kindergarten year (see Table 4). The Latin-American children most frequently utilized a moral approach while the Asian-American children employed this method the least. The gender generally did not differ in their social-emotional competence at the end of the kindergarten year (see Table 4). However, the most dysregulated aggression was employed by the male children (Table 4).

**Table 4:** Ethnic and gender specificity at the end of the kindergarten year (multivariate two factorial analysis of variance).

	**F (df = 1;2)^d^**		**P (F)**				**Wilks-Lambda^c^**		**p**	
**Variable**	**ethnic**	**gender**	**ethnic**	**gender**	**η^2^_ethnic_^a^**	**η^2^_gender_^b^**	**ethnic**	**gender**	**ethnic**	**gender**

							2.19	0.22	**.04**	.11
**Empathetic relation**	2.15	0.54	.15	.47	.03	.02				
**Avoidance strategies**	1.33	0.16	.26	.69	.01	.01				
**Moral themes**	5.09	2.59	**.03**	.11	.05	.07				
**Interpersonal conflict**	0.09	0.34	.93	.56	<.01	.03				
**Dissociation codes**	0.19	1.29	.67	.26	<.01	.01				
**Dysregulated aggression**	0.00	5.50	.95	**.02**	<.01	.08				
**Narrative emotions**	2.79	0.74	.10	.40	.04	.01				

^a, b^Effect sizes for the ethnic groups and gender specific change; ^c ^Exact test; ^d ^corrected model; Ethnic*gender interaction not significant

The change in the social-emotional competence and its ethnic specificity: Herefore, a three-factorial (time, gender, ethnic group) univariate analysis of variance with repeated measurement points was calculated separately for each scale of the MSSB for the MSSB-codes at the beginning and at the end of the kindergarten year (Table 5). Greenhouse-Geisser correction was applied to account for violation of the sphericity assumption.

The data showed no significant changes in the MSSB-codes with the exception of the expressed emotions in the narratives. The children expressed more emotions at the end of the kindergarten year than at the beginning (see Table 5).

Second, the ethnic specificities in the change of social-emotional competence were examined (Table 5). For the avoidance strategies, there was an ethnic group-by-time interaction effect present. The avoidance strategies of the Asian-American and Latin-American children developed differently: Those of the Asian-American children increased, and the Latin-American children's decreased.

Third, the gender specificities in the change of social-emotional competence were analyzed (Table 5). There were no developments in a gender-specific way over the course of the kindergarten year.

**Table 5:** Changes in social-emotional competence.

	**F (df = 1; 2)^c^**						**p(F)**								
				
	**time**	**ethnic**	**gender**	time* ethnic	time* gender	time* ethnic*gender	**time**	**ethnic**	**gender**	time* ethnic	time* gender	time* ethnic*gender	**η^2^_time_**	**η^2^_ethnic_^a^**	**η^2^_gender_^b^**
**Empathetic relation**	1.70	1.05	1.37	1.48	0.00	1.30	.20	.31	.25	.23	.98	.26	.03	.02	.03
**Avoidance strategies**	1.03	<.01	0.14	4.85	0.00	0.17	.31	.99	.71	**.03**	.97	.68	.02	<.01	<.01
**Moral themes**	0.11	7.82	6.30	0.00	0.50	.30	.74	**.01**	**.02**	.99	.48	.59	<.01	.13	.11
**Interpersonal conflict**	2.52	1.35	.31	2.16	0.00	5.17	.12	.25	.58	.15	.99	**.03**	.05	.03	.01
**Dissociation codes**	0.56	0.88	2.87	0.63	1.08	1.03	.46	.35	.10	.43	.30	.32	.01	.02	.05
**Dysregulated aggression**	1.43	0.04	6.20	0.00	0.06	.67	.24	.85	**.02**	.96	.81	.42	.03	<.01	.11
**Narrative emotions**	12.95	1.99	0.47	0.79	0.17	.00	**.00**	.16	.50	.38	.68	.94	.20	.04	.01

A three factorial (time, gender, ethnicity) univariate analysis of variance with repeated measurement.

^a, b^Effect sizes for the time, ethnic and gender specific change; ^c ^Greenhouse-Geisser corrected; (M and SD see Table 1); Ethnic*gender interaction not significant

Fourth, there was a significant time*ethnic*gender interaction effect tor interpersonal conflict. The Asian Americans, girls as well as boys, increased the implementation of interpersonal conflicts in contrast to the decrease in the Latin-American children. The male children do it more intense than the girls do.

## Discussion

The core findings of this study were:

1. The two ethnic groups in the classroom differed in moral reasoning at the beginning of the kindergarten year. A moral approach was utilized the most by the Latin-American children and the least by the Asian-American children.

2. The two ethnic groups did also differ in moral reasoning at the end of the kindergarten year.

3. At the beginning of the kindergarten year the gender groups differed in moral themes as well as in dysregulated aggression and dissociation codes. The boys showed more aggressive behavior than the girls, who displayed more moral reasoning. At the end of the kindergarten year the gender groups differed in dysregulated aggression since the boys implemented it more often than the girls.

4. The two ethnic groups developed differently concerning avoidance strategies and displayed at the end more expressed emotions in their narratives than at the beginning of the kindergarten year. The Asian-American children increased and the Latin-American children decreased the avoidance of conflicts over the course of the kindergarten year.

At the beginning of the kindergarten year (Hypotheses 1), compared to the Asian-American children, the Latin-American children more frequently applied moral themes to solving the stories. Using questionnaires and observations, Samples and colleagues were also able to observe more moral reasoning in Latin-American children than in their African-American counterparts [33]. This ethnic specificity might be explained by the strong Catholic background and the emphasis on a moral value system typical of their upbringing as reported in the literature [47]. Collectivism and obligation to the family was found to be prevalent in Mexican-American children and might explain the high usage of moral reasoning in this ethnic group [19].

At the end of the kindergarten year (Hypotheses 2), the ethnic differences in moral reasoning were still present. The results did not support the hypothesis that not only the ethnic background shapes moral reasoning or rather the value system during the kindergarten year. The results clearly showed that during one year the moral value system can not be enriched by experiences in the kindergarten. However, these data did not specify to which extend the moral value system was focused on in the kindergarten classroom. Since moral development is important for the development of social-emotional competence, the moral value system need to be addressed already in kindergarten. Based on the present data it can be further assumed that the "main effect model" might be independent of the ethnicity [26]. The ethnic-specific usage of aggression was not accompanied by ethnic-specific implementation of empathy and expressed emotions. However, the ethnic-specificity of the model has to be evaluated in more detail before conclusions can be drawn.

Concerning aggression, gender specificity was prevalent (Hypotheses 3). Hereby, the male children most frequently utilized dysregulated aggression compared to the female children. Gender differences concerning aggression are already well documented in the literature [48-51]. In addition to the ethnic differences at the beginning of the kindergarten year, female children used more moral reasoning than the male children. Also, the female children of the Wittenberg's study showed more moral reasoning, fairness and empathy than their male counterparts [52].

In a pre-post comparison (Hypotheses 4) the children of the two ethnic groups showed a significant increase in expressed emotions in the doll play narratives. As found in the literature, the exposure to prosocial peers – in this study the high amount of expressed emotions in the Latin-American children – was related to improved social behavior in antisocial peers one year later [8]. To detect changes in the other areas of social competences the observation phase of one year as well as the exposure of six hours per day had to be expanded. Furthermore, ethnically specific development took place exclusively in the avoidance strategies. The use of avoidance strategies increased in the Asian-American children whereas it decreased in the Latin-American children. The same contrasting development took place in tendencies in interpersonal conflicts. This decrease in avoidance strategies and interpersonal conflicts in Latin-American children replicated the process that the level of negative behavior decreased in children with initially higher levels when exposed to their more prosocial peers [3].

The results of this study rely on the observed doll play behavior, which is a good predictor for classroom behavior [41]. Even so, an additional observation of the classroom behavior would be helpful for drawing conclusions on child behavior in general. One limitation to this study was the relatively small sample size which was considered in the calculations. This can be explained by the distribution of ethnic groups in this particular Oakland district. A second limitation was the lack of information concerning the year of immigration and socio-demographic information of these children such as family situation, country of birth and religion. Information on the year of immigration would have been helpful to further specify the degree of integration and to weight the ethnic differences. Presumably, there may have been a varied degree of integration in this sample as the children came from a natural setting. Furthermore, the cultural background and its influence on child-rearing might possibly be linked to the religion practiced by the individual family group. Also, income and family situation could be of help to further understand the ethnic differences better. Respectively, one needs to proceed with caution when trying to transfer these conclusions to children of other ethnic groups or countries.

The strengths of this study are: Until now there have been only a few studies examining the effects of multi-ethnic-classrooms on child development. A strength of this study is the chosen setting. The kindergarten was located in a high risk area and attended exclusively by the children of families with an immigration background. In addition, the children were observed twice: at the beginning and at the end of their kindergarten year. The social-emotional competence was observed by an independent person who did not belong to the kindergarten staff.

As a recommendation for future studies, the design for examining social-emotional competence should definitely include doll play and observations of classroom behavior observation in multi-ethnic and non-multi-ethnic classrooms with large sample sizes. In a longitudinal design, additional development in the social-emotional competence of the different ethnic groups might be found. As an additional focus, school performance as well as language skills should be contained as well. It would be essential to also include an aggression prevention program as well as a program to increase social-emotional competence and their longitudinal outcome. There are more and more kindergartens where for example Spanish or Chinese are the classroom language for one ethnicity. The development of children attending such a classroom needs to be compared to children of multi-ethnic classrooms. This should be followed by a study of the effect of the exposure of multi-ethnicity during the children's later developments.

In summary, after taking the gender effects into account a specific ethnic diversity in social-emotional competence did exist in the particular sample. Considering the different highs and lows in the competencies of the ethnic groups in this study leads to the assumption that a natural model learning and imitation process did occur, e.g. the large amount of expressed emotions of the Latin-American children presented a model for the less emotionally expressive Asian-American children. Therefore, model learning as well as learning social-emotional competence in the early years is essential to prevent problematic behavior in multi-ethnic classrooms. These multi-ethnic classrooms build an environment with until now unknown effects on the child development. The more knowledge of early learning can be assimilated, the easier it will be to spot and prevent problematic behavior. Based on this knowledge programs can be developed to prevent and eliminate problematic behavior. In order to get to the early roots of problematic behavior these programs should especially focus on moral development and empathetic competence [53,54].

## Competing interests

The authors declare that they have no competing interests.

## Authors' contributions

KP did the first and final draft of the manuscript and critically revised it for the intellectual content. AvW has given final the approval of the version to be published. UH substantially contributed to the analysis and the interpretation of data. MC and PJ were responsible for the general supervision of the research group. They substantially contributed to the conception and the design of the study as well as the acquisition of the funding. All authors read and approved the final manuscript.
